# Polyphenols profile of pomegranate leaves and their role in green synthesis of silver nanoparticles

**DOI:** 10.1038/s41598-020-71847-5

**Published:** 2020-09-09

**Authors:** Noha Swilam, Khaled A. Nematallah

**Affiliations:** Department of Pharmacognosy, Faculty of Pharmacy, The British University in Egypt, El Sherouk City, Suez Desert Road, P.O. Box 43, Cairo, 11837 Egypt

**Keywords:** Nanoparticles, Secondary metabolism

## Abstract

The current study reports on polyphenols profile of pomegranate leaves (PL) *Punica granatum* grown in Egypt and exhibiting their role in development of an eco-friendly method of green synthesis of silver nanoparticles (AgNPs). PL aqueous alcohol extract was fractionated, the major phenolic compound was isolated from the polyphenols rich fraction (ethyl acetate fraction) and identified by conventional and spectroscopic methods of analysis as ellagic acid. Furthermore, the fraction was standardized and analysed using UPLC-PDA-UV and LC–MS-MS techniques revealing tentative identification of 23 polyphenolic compounds, quantifying ellagic acid as 43.14 ± 0.57 μg/mg of the fraction. AgNPs were successfully synthesized with the aid of polyphenols rich fraction. This is the first report revealing the systematic optimization of the green synthesis process using various independent variables. AgNPs were characterized by adopting UV–Vis spectroscopy, FTIR, XRD, and SEM, which revealed strong SPR band within average of λ _max_ 425 nm and polygonal shaped nanoparticles of 26.22 nm size, respectively. The antimicrobial efficacies of AgNPs and polyphenols rich fraction were tested against Gram-positive bacteria (*Bacillus subtilis, Staphylococcus aureus,* and *Sarcina lutea*), Gram-negative bacteria (*Salmonella paratyphi*, *Escherichia coli,* and *Pseudomonas aeruginosa*) and fungi (*Candida albicans*). AgNPs showed a concentration-dependent activity against all the tested microorganisms.

## Introduction

Nanotechnology is a fast-growing area of science with beneficial roles to various fields of health sciences such as food industry, health care, biomedicals, water treatment, and cosmetics^1,2^. Nanoparticles (NPs) are one of the excellent findings of nanotechnology to solve the day to day issues of the current world. One of the most important metallic NPs is silver nanoparticles (AgNPs); because of their chemical stability, good conductivity, antibacterial, antiviral, antifungal and cytotoxic activities^3–5^.

The effective conductivity of AgNPs has expanded their applications in a wide array of products such as electronic devices, inks, adhesives, pastes and in controlling microbial growth and infections^6,7^, which has also made them eco-friendly . Additionally, they are being added to skin creams, wound dressings, antiseptic fabrics and sprays on account of their wide antiseptic properties^8^. Moreover, it was proved that AgNPs in addition to other materials such as hydroxyapatite or yttrium oxide have the ability to purify water from the heavy metals and several organic matters^9,10^. The size of the global market of AgNPs increased markedly, the global market revenue grows from 1.1 billion USD in 2016 to expected value of 3.0 billion USD by 2021^11^.

Generally, AgNPs are synthesized by different physical and chemical processes that are relatively expensive and utilize different toxic and hazardous chemicals. One of the approaches to get rid of utilization of such hazardous chemicals is the bio/green synthesis process, in which biological entities like microorganisms or plant extracts are used in the production of the nanoparticles^12^.

Plant metabolites are considered as reducing agents that reduce silver ions into silver metal, and a capping agent that stabilizes the size and shape of the produced nanoparticles^13^. Although several groups of plant metabolites such as carbohydrates, proteins, alkaloids and terpenoids were reported in the green synthesis process of metallic nanoparticles, polyphenol compounds including flavonoids are the major phytochemicals that are included in such a process, due to their powerful reducing properties and high stability of the synthesized nanoparticles^14^.

*Punica granatum* (Pomegranate) is a plant whose fruits are largely consumed all over the world. It is well known for its richness in polyphenols^15^. Apart from the fruits, other plant parts such as peels, leaves, flowers are included in different food preparations like beverage tea, jams, jellies, sauces and salad dressings^16^. Different plant parts like seeds, peels and leaves were reported before in the green synthesis of AgNPs^17–20^. The present study aimed at profiling the polyphenols of pomegranate leaves (PL) grown in Egypt and proving their role in the green synthesis process of AgNPs. This incorporates extraction, fractionation, standardization, and polyphenols profiling of PL extract adopting UPLC-PDA-UV and LC–MS–MS methods. Besides, isolation and structural elucidation of the main polyphenol component. Moreover, facile green synthesis of AgNPs with the aid of pomegranate polyphenols, characterization, and investigation of its antimicrobial activity.

## Results

### Extraction and standardization of the extract

One of the main goals of this study is to prepare a standardized polyphenols rich fraction from the aqueous alcohol extract of pomegranate leaves (PL). The spectrophotometric assay of the total polyphenols and flavonoids contents showed that ethyl acetate fraction was the richest fraction in polyphenols and flavonoids with 586.25 mg gallic acid equivalent (GAE)/g fraction and 194.67 mg rutin equivalent (RE)/g fraction, followed by the crude extract with 396.75 mg GAE/g extract and 125.60 mg RE/g extract, while the least amount was found in *n*-butanol fraction with 353.00 mg GAE/g fraction and 91.30 mg RE/g fraction.

The ethyl acetate fraction was subjected to automated flash chromatography to isolate its major phenolic compound, that was isolated as buff colored powder (342 mg) with ^1^H-NMR (DMSO-d_6_, 400 MHz, δ = ppm), 7.47 (2H, *s*, H-5 and H-5′), and ^13^C-NMR (DMSO-d_6_, 100 MHz, δ = ppm), 112.80 (C1), 136.84 (C2), 140.17 (C3), 148.60 (C4), 110.65 (C5), 108.03 (C6), and 159.64 (C7). The UV–Visible spectrum of that compound exhibited λ_max_ at 253.7 nm and 367.5 nm. The received spectral data were consistent with those reported for ellagic acid^21^.

**Figure 1:**
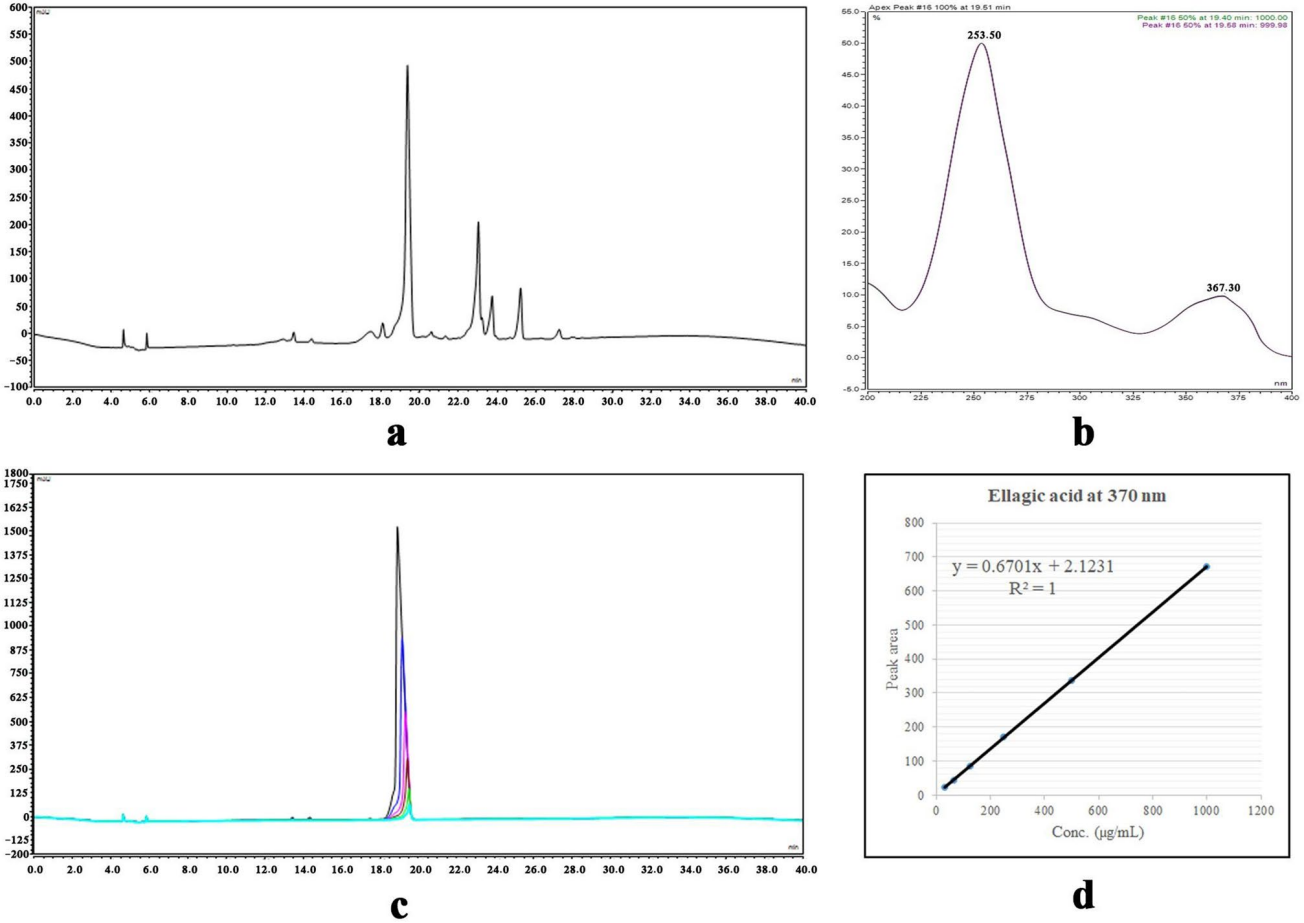
Standardization of the ethyl acetate fraction, (**a**) UPLC Chromatogram of the ethyl acetate fraction, (**b**) UV spectrum of the peak at Rt = 19.4 min, (**c**) UPLC Chromatogram of the isolated ellagic acid at different concentrations, (**d**) Calibration curve of the isolated ellagic acid.

The ethyl acetate faction was standardized by UPLC technique, the total ion current chromatogram of the fraction was recorded at 370 nm (Fig. 1a), that showed a major peak (44.54%) at Rt = 19.4 min, with UV spectrum, that exhibited λ_max_ at 253.5 nm and 367.3 nm (Fig. 1b) that was identified as ellagic acid by co-chromatography with the previously isolated compound. A calibration curve of the isolated ellagic acid was established on the same UPLC device with the same conditions and parameters (Fig. 1c). The curve equation was found to be y = 0.6701x + 2.1231 and *R*^*2*^ = 1 (Fig. 1d). It was found that each mg of the fraction contains 43.14 ± 0.57 μg ellagic acid.

### LC–MS–MS analysis of ethyl acetate fraction of PL

It is the first time to report the polyphenols profile of the ethyl acetate fraction (the polyphenols rich fraction) using a UPLC–PDA–ESI–MS method (Table 1). Twenty-three compounds were tentatively identified; most of them (11 compounds) were found to be phenolic acids and their derivatives. Furthermore, 8 compounds were found to be tannins; 7 of them were hydrolysable tannins while only one compound was a condensed tannin. Moreover, 3 anthocyanins and 1 flavonoid derivative were detected.

**Table 1:** LC–MS–MS data of the tentatively identified compounds in the ethyl acetate fraction.

Peak	R_t_ (min)	Identified compounds	UV–Vis (λ_max_)	[M−H]^−^ (m/z)	Fragment ions (m/z)	Percentage (%)	Refs
1	3.4	Galloyl-HHDP-hexoside	333, 260	633.2	301, 249	0.54	^15,52,53^
2	4.2	Galloyl-HHDP-DHHDP-hexoside (Granatin B)	365, 274	951.2	933, 301	0.27	^15,53^
3	4.8	Gallic acid	274	169.2	125	1.25	^15,52–54^
4	4.82	Castalagin derivative	275	965.2	301	0.89	^15^
5	5.3	Bis-HHDP-hexoside	320, 256	783	765, 481, 301	0.21	^13,47,48^
6	5.6	Digalloyl-HHDP-hexoside	375, 268	801.1	649, 301	0.12	^13^
7	5.9	Cyanidin pentoside	515, 278	417.1	287	0.12	^49^
8	6.6	HHDP-hexoside	267	481.2	301, 275, 229	0.16	^13,47,48^
9	6.9	Ellagic acid	367, 275	301	229, 185	2.89	^13,47,48^
10	7.3	Procyanidin dimer	377, 285	577.2	441, 289	0.35	^49^
11	7.67	Galloyl hexoside	375, 265	331.1	271, 269, 125	0.28	^13,47^
12	9.7	Dimethyl ellagic acid glucuronide	365, 270	505.1	329, 314	0.08	^48^
13	10.5	Ellagic acid deoxyhexoside	366	447.3	301	0.19	^13,47^
14	15.3	Ellagic acid dihexoside	319, 286	625	425, 301	0.08	^13^
15	25.7	Ellagic acid hexoside	361, 252	463.2	301, 271	0.32	^13,47^
16	26.2	Feruloyl coniferin	225	517.1	337, 271	0.62	^47^
17	27.3	Galloyl-HHDP-gluconate	375, 265	649.2	497, 301	1.83	^13,47^
18	39.2	Coumaroyl hexose	315	325.1	163	0.13	^49^
19	39.3	Caffeoyl quinic acid	325, 210	353.1	191	0.12	^13^
20	39.8	Dicaffeoyl quinic acid	325, 220	515.4	353, 191	0.09	^49^
21	40.58	Pelargonidin	369, 229	271.1	181	0.05	^49^
22	41.32	Delphindin dihexoside	519, 277	627	303	0.31	^13,49^
23	43.32	Phloretin-hexoside (Phlorizin)	287	435.4	167	0.02	^47,49^

### Green synthesis of AgNPs and its optimization

Firstly, crude extract as well as the ethyl acetate and *n*-butanol fractions at different concentrations were preliminary tested in the green synthesis process. It was found that the AgNPs that were synthesized by the aid of the ethyl acetate fraction was the best, in terms of its SPR bands (λ_max_ = 417 nm) and particle size (34 nm).

**Figure 2:**
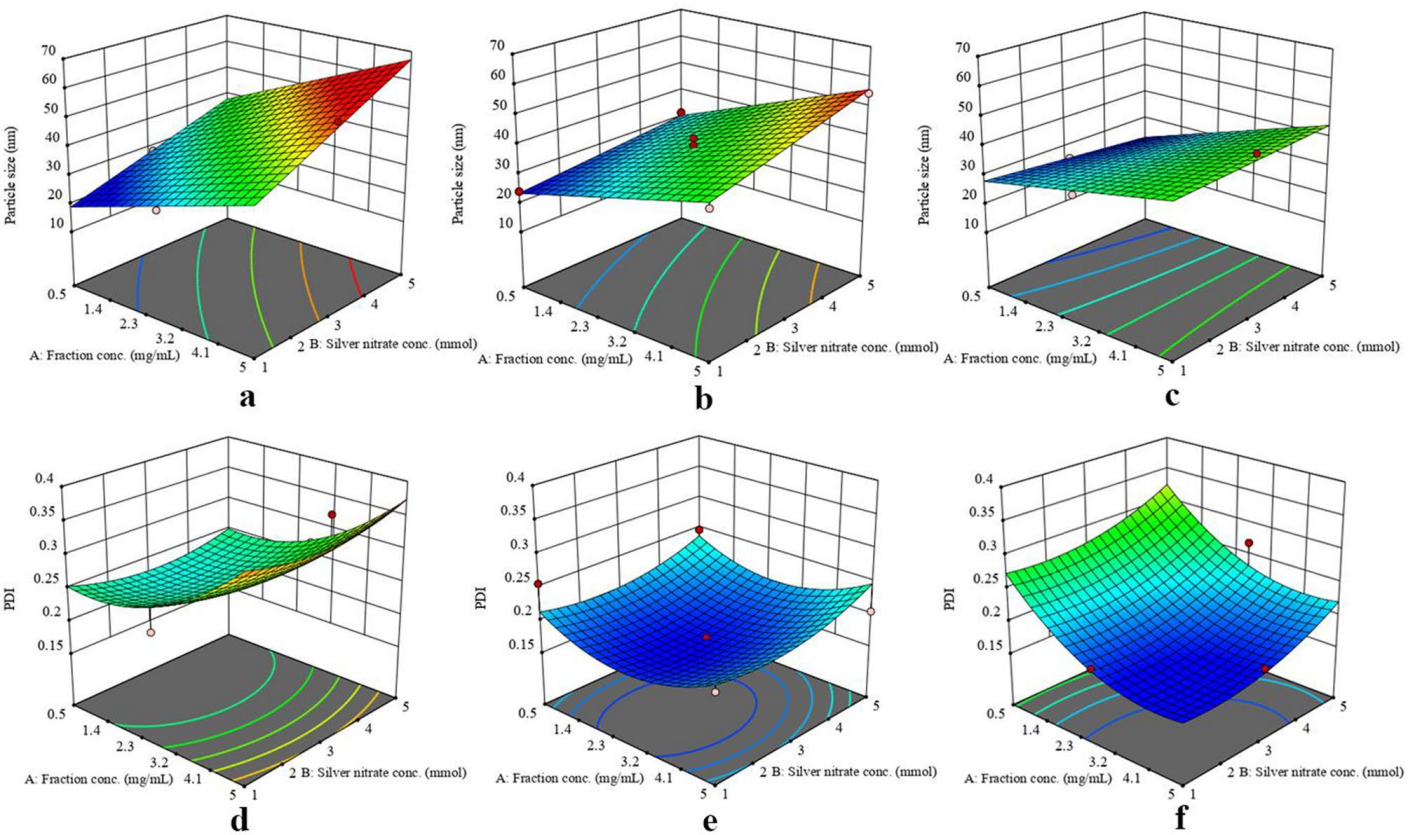
3D response surface plots for particle size responses at 60 °C (**a**), 70 °C (**b**) and 80 °C (**c**) and PDI responses at 60 °C (**d**), 70 °C (**e**) and 80 °C (**f**).

Systematic optimization was carried out to find the best conditions for synthesis of AgNPs by PL for the first time. Fourteen experimental trials were carried out and responses were recorded (Table 2), the obtained data were subjected to the chosen models followed by performing fitting analysis using various statistical parameters. After data modelling, two polynomial equations were generated (Eqs. 1 and 2). Those equations proved that there is an interaction and curvature effect for the two variables analysed (particle size and PDI). Furthermore, good fitting of data within the selected models was illustrated in the 3D plots of responses (Fig. 2)

1 $$ {\text{Particle}}\;{\text{size}} = - {36}.{265} + {9}.{\text{289A}} + {23}.0{\text{31B}} + 0.{8}0{\text{5C}} + 0.{45}0{\text{AB}} - 0.0{\text{84AC}} - 0.{3}0{\text{8BC}} $$

2 $$ {\text{PDI}} = 0.{166} + 0.00{\text{22A}} + 0.0{\text{146B}} - 0.0{\text{334C}} + 0.00{\text{28AB}} - 0.0{\text{568AC}} + + 0.0{\text{135BC}} + 0.0{\text{398A}}^{{2}} + { + }0.0{\text{215B}}^{{2}} + 0.0{5}0{\text{5C}}^{{2}} $$

where A is the fraction concentration (mg/mL), B is the silver nitrate concentration (mMol) and C is the temperature (°C).

**Table 2:** Design matrix showing trial runs performed for optimization of green synthesis of silver nanoparticles by the aid of pomegranate leaves extract (ethyl acetate fraction) using Box–Behnken Design.

Run	Factor 1 A: fraction conc. (mg/mL)	Factor 2 B: silver nitrate conc. (mMol)	Factor 3 C: temperature (ºC)	Response 1 Particle size (Nm)	Response 2 PDI
1	2.75	5	80	31.4	0.269
2	0.5	3	80	25.2	0.231
3	2.75	1	60	28.1	0.234
4	5	3	80	45.1	0.175
5	5	3	60	55.8	0.395
6	2.75	1	80	33.3	0.18
7	5	5	70	54.5	0.204
8	0.5	3	60	28.3	0.224
9	2.75	5	60	50.8	0.269
10	0.5	5	70	31.6	0.247
11	2.75	3	70	40.8	0.168
12	0.5	1	70	24.1	0.256
13	5	1	70	38.9	0.202
14	2.75	3	70	38.3	0.164

Regarding the particle size response, ANOVA results showed that the 2FI model is significant with P-value < 0.05, where A, B, and C are significant model terms. *R*^2^ = 0.9733, with lack of fit F-value of 1.92 that implies non-significant lack of fit relative to the pure error. The quadratic model for the PDI response was found to be non-significant, with a significant lack of fit F-value of 455.14, this could be attributed to closeness of the PDI results to each other within the tested ranges, as they are all in the acceptable range below 0.400 (Table 3).

**Table 3:** Antimicrobial activity of the synthesized silver nanoparticles at different concentrations, ethyl acetate fraction and silver nitrate solution, tested by well diffusion method.

Organism	Inhibition zone diameter (mm)						
	Silver nanoparticles					Ethyl acetate fraction 0.45 mg/100 µL	Silver nitrate solution 0.45 mg/100 µL
	0.05 mg/100 µL	0.15 mg/100 µL	0.25 mg/100 µL	0.35 mg/100 µL	0.45 mg/100 µL		
*Bacillus subtilis*	12	16	18	20	23	19	31
*Staphylococcus aureus*	11	13	14	15	17	22	23
*Sarcina lutea*	13	16	17	19	19	34	27
*Salmonella paratyphi*	10	13	15	15	18	0	20
*Escherichia coli*	12	13	14	16	18	0	22
*Pseudomonas aeruginosa*	11	11	12	15	16	0	22
*Candida albicans*	0	11	14	16	17	0	27

The optimum conditions of synthesis of AgNPs using the ethyl acetate fraction was suggested by numerical optimization where desirability function value closer to 1. The target for each response variable was minimization to the least possible value. The optimum conditions were found to be fraction concentration of 0.83 mg/mL, silver nitrate concentration of 1.15 mMol and at 67.5 °C, with predicted values; particle size (24.1 nm) and PDI (0.202).

### Characterization of the prepared AgNPs at the optimum conditions

#### UV–Visible spectrophotometry

In order to monitor the formation of AgNPs, their SPR bands were recorded at different time intervals (30, 60, 90, 120 and 150 min) during the green synthesis process. In all measurements, AgNPs exhibit strong SPR band within average of λ _max_ 425 nm. The intensity of the absorbance increases with time, however, the lowest λ_max_ was shown after incubation for 60 min (438 nm), indicating nanoparticles with smaller diameter^22^.

#### FTIR analysis

**Figure 3:**
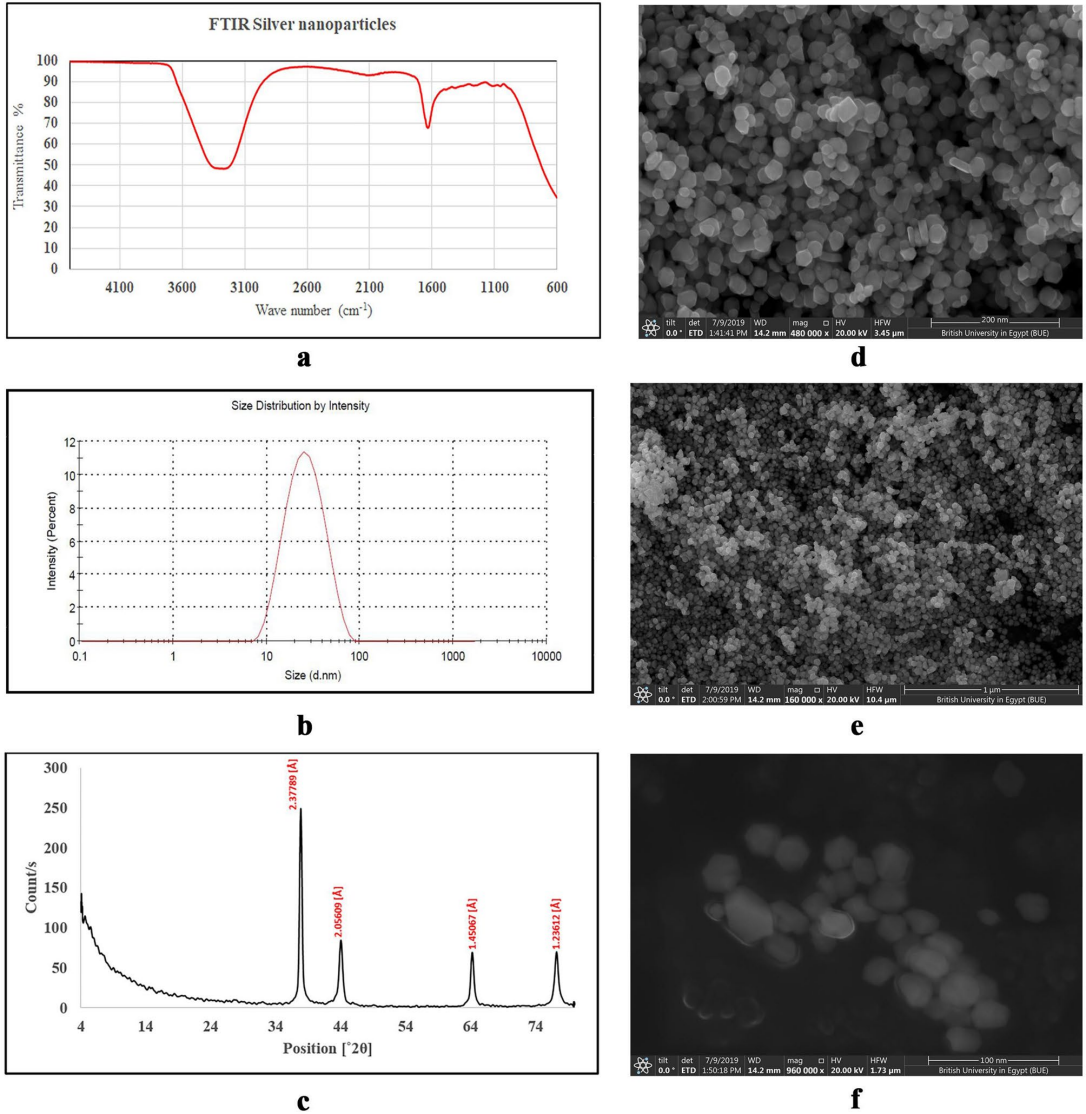
Characterization of the synthesized silver nanoparticles (AgNPs) at the optimum conditions, (**a**) FTIR spectrum of AgNPs, (**b**) size distribution by intensity using light scattering technique, (**c**) XRD pattern, (**d**) SEM image of AgNPs at magnification power × 480,000, (**e**) SEM image of AgNPs at magnification power × 160,000, (**f**) SEM image of AgNPs at magnification power × 960,000.

FTIR analysis was carried out to determine the important functional groups on the surface of the nanoparticles (Fig. 3a). IR spectrum showed strong peaks at 1635 cm^−1^ and 3,300 cm^−1^ corresponding to presence of C = C and phenolic –OH respectively. Hence, the obtained results indicate involvement of pomegranate phenolic compounds in the reduction of silver ions and stabilization of the size and shape of the nanoparticles.

#### Particle size analysis

The average diameter of the synthesized nanoparticles was determined by light scattering technique and found to be 26.22 ± 0.63 nm with PDI 0.189 (Fig. 3b).

#### XRD analysis

The crystalline nature of the AgNPs was confirmed by X-ray crystallography. The XRD pattern of the synthesized AgNPs is shown in (Fig. 3c). The diffracted intensities were recorded from 2θ of 4° to 2θ of 80°. The XRD pattern showed four strong Bragg reflections at 37.84°, 44.00°, 64.20° and 77.17°, with relative intensities of 100%, 32.26%, 27.28% and 26.63% respectively. Those four peaks correspond to the planes of (1 1 1), (2 0 0), (2 2 0) and (3 1 1) respectively, which could be indexed according to the facets of face centred cubic crystal structure of silver^23^. Moreover, the nature of crystals was confirmed to be silver by the interplanar spacing (d_calculated_) values of 2.378, 2.056, 1.451 and 1.236 Å for (1 1 1), (2 0 0), (2 2 0) and (3 1 1) planes respectively that are matched with standard silver values. The average crystalline size is calculated using Debye–Scherrer formula,

$$   {\text{D}} = \frac{{{\text{k}}\uplambda }}{{\beta \cos \uptheta }}  $$

where D is the average crystalline size of the nanoparticles, k is the geometric factor (= 0.9), λis the wavelength of the X-ray radiation source in Å and β is the angular full-width at half maximum (FWHM) in Rad of the XRD peak at the diffraction angle θ. The calculated average crystalline size of the AgNPs is ~ 32 nm.

#### Scanning electron microscopy

The SEM analysis was performed to determine the exact shape and size of the synthesized nanoparticles (Fig. 3). The figures show that the average diameter of the particles was from 20 to 40 nm, and the shape was nearly polygonal. Size range and shape distribution was nearly equal all over the sample (Fig. 3e). The high magnification photo (Fig. 3f) illustrated the capping of bioactive compounds from the ethyl acetate fraction on the surface of the nanoparticles.

#### Antimicrobial activity

The antimicrobial activity of AgNPs, polyphenols rich fraction and silver nitrate were investigated. The results shown in table (3) revealed that the fraction exhibited antimicrobial activity against *Bacillus subtilis*, *Staphylococcus aureus* and *Sarcina lutea*, while other microorganisms were resistant to the fraction, the highest activity was against *Sarcina lutea* with inhibition zone diameter 34 mm. The highest antimicrobial activity of AgNPs was found to be against *Bacillus subtilis,* the rest of tested microorganisms have more or less equal sensitivity to the synthesized AgNPs with a concentration dependent manner. Silver nitrate solution showed the highest antimicrobial activity against all the tested microorganisms except *Sarcina lutea*.

## Discussion

Currently, green synthesis is gaining a great interest in the production of metal nanoparticles, due to their ease of synthesis, eco and environmentally friendly, and utilization of plants waste products^24^.

Aqueous extract of PL was found to have a good potential in the production of AgNPs with anticancer activity on human cervical cancer cells, liver cancer cells (HepG2) and antidiabetic activity^18,20,25^. Based on the reported data, there was a need for preparation of a standardized phytochemically analysed fraction and correlate that action with its components. To achieve that goal, the aqueous alcohol extract of PL was fractionated using *n*-hexane, methylene chloride, ethyl acetate and *n*-butanol. The crude extract as well as the different fractions at different concentrations were preliminary tested for their ability to synthesize AgNPs with good characteristics. The comparison between the outcome of each experiment was based on its SPR band as the best nanoparticles would have the lowest λ_max_ with the highest intensity^22^. Moreover, spectrophotometric determination of total polyphenols and flavonoids were carried out for the previous samples. It was found that the best characteristics of the AgNPs were synthesized by the aid of the polyphenols rich fraction (ethyl acetate fraction).

The ethyl acetate fraction was standardized using UPLC-PDA-UV method with reference to the major phenolic compound that was isolated from the fraction itself; which was found to be ellagic acid. The fraction was further analysed by an LC–MS–MS method that revealed tentative identification of 23 polyphenols for the first time in the leaves of pomegranate grown in Egypt (Table 1).

Many studies before mentioned the role of plant polyphenols in the green synthesis of AgNPs. For example, the phenolic extracts obtained from Bilberry and Red Currant waste extracts were found to produce AgNPs with a hydrodynamic diameter of 25–65 nm^26^. Jigyasa and Rajput (2018) promoted the green synthesis of AgNPs for colorimetric detection of melamine in milk using different bio-polyphenols such as rutin and curcumin^27^. More specifically, gallic acid and quercetin mediated the synthesis of silver and bimetallic (silver and selenium) nanoparticles with antimicrobial and antitumor activities^28,29^. More importantly, ellagic acid was not only has the capability of reducing silver ions, resulting in the formation of AgNPs, further, the AgNPs in the presence of ellagic acid were highly effective as antibacterial agents, this finding support our results^30^.

From the optimization 3D plot diagrams shown in (Fig. 2), it was deduced that there is an increase in the particle size in correlation to the increase in concentration of the ethyl acetate fraction and/or silver nitrate, while there is an inverse correlation with temperature. It was reported that increasing in the silver nitrate concentration increases the particle size, as they were easy to aggregate into larger particles, due to the rise of the collision frequency with an increasing of silver salt content^31^. Moreover, the temperature was found to have a significant effect on the formation, shape, size and size distribution of particles^32^. Liu et al. (2017) found that the size of AgNPs was slightly increased from 70 to 80 °C, while it decreased sharply from 80 to 90 °C, they concluded that the decrease of size at high temperature is a result from the sharply increased nucleation rate constant instead of the decreased growth rate constant^33^. Although, the statistical model of the PDI was found to be not significant, it is obvious from the diagrams (Fig. 2d–f) that PDI has an optimum range of temperature to be minimized, less or more than that range, PDI will increase.

A characterization profile was carried out for the AgNPs obtained from the optimized experiment. Particle size was analysed by light scattering technique and SEM, the results were relatively in the same range and in consistence with the theoretically calculated particle size from the polynomial equation obtained after data modelling and the Debye–Scherrer formula obtained from the XRD analysis. In the current study, the particle size of the synthesized AgNPs by the aid of the polyphenols rich fraction was found to be relatively smaller than those reported before in literature which obtained from crude aqueous extracts of different organs of pomegranate^34^, the range of particle sizes of AgNPs prepared by crude aqueous extract of pomegranate leaves was found to be 35–70 nm^18,20^.

The UV–Visible spectral analysis showed λ_max_ in the range that is similar to those reported before for AgNPs synthesized by pomegranate leaves^18,20^. Similar type of analysis with nearly the same range of results was carried out before in recent studies of synthesis of AgNPs using extracts of different plants, such as *Allium cepa* (onion), *Parkia speciose*, and *Salvia hispanica*^35–37^.

FTIR analysis confirmed the role of pomegranate phytochemicals as reducing and stabilizing agents for the AgNPs. The spectrum revealed presence of C=C stretching vibrations of the phenolic compounds and phenolic hydroxyl groups. Similar findings were obtained from the AgNPs prepared by *Diospyros lotus* and *Thunbergia grandiflora* extracts^38,39^. As mentioned before, ellagic acid is the major compound in the PL, it was reported that ellagic acid which is the major antioxidant component in the combined peels extract of pomegranate, orange, banana and apple is responsible for the synthesis of AgNPs, as it has an easy electron loosing capacity which results in formation of H^+^ radical, that reduces the size of silver to nano size^40^.

XRD analysis confirmed the cubic nature of the AgNPs (Joint Committee on Powder Diffraction Standards; JCPDS no. 04-0783), the results are consistent with previous studies, reporting similar diffraction peaks^20,23^. Size and nature of AgNPs was confirmed by the high magnification using SEM technique, which also confirmed presence of an organic shell around the nanoparticles.

Antimicrobial activities of the synthesized AgNPs, polyphenols rich fraction and silver nitrate solution were tested against different Gram positive, Gram negative bacteria, and fungi. The majority of the tested microorganisms had resistance to the fraction. On the other hand, AgNPs showed antimicrobial activity against all the tested microorganisms. As silver nitrate solution exhibited strong antimicrobial activity in contrast to the fraction, that could rationalize the antimicrobial characters of the AgNPs by presence of silver metal rather than the coating organic material. The antimicrobial activity of silver metal is reported for a wide range of over 650 microorganisms from different classes such as viruses, gram positive and gram negative bacterial, and fungi^41^. The bactericidal effect of silver nitrate was reported before^42,43^, it was found that at lower concentrations it induced synthesis of AgNPs, whereas at higher concentrations it induced cell death^44^. The higher antimicrobial activity of silver nitrate solution than AgNPs against all tested bacteria is highly supported with those reported by Li et al.^45^ and Kedziora et al.^46^.

It was reported that different extracts of PL had moderate antimicrobial activity against various human pathogens^47^. Pomegranate peels extract was also found to have *in-vitro* and *in-vivo* antibacterial activity against different strains of *Salmonella*^48^.

In conclusion, in this study twenty three polyphenolic compounds were tentatively identified by LC–MS–MS techniques in ethyl acetate fraction. This fraction was standardized by UPLC–PDA–UV method with reference to its major compound ellagic acid. AgNPs were biosynthesized using polyphenol rich fraction of PL in a simple one step process. The production of AgNPs was correlated to the phenolic content of PL and FTIR results confirmed this finding. Systematic optimization of AgNPs using PL proved the direct correlation between the particle size with silver nitrate and fraction concentrations, and inverse correlation with the temperature. AgNPs were found to have an average size of 26.22 nm size, polygonal structure, absorbance maxima at 425 nm and the presence of biocompatible capping over it. The antibacterial activity of the synthesized AgNPs on tested organisms further confirms the activity to the silver metal rather than the polyphenols adsorbed on the surface of the AgNPs. Finally, pomegranate polyphenols could provide promising opportunities in silver nanoparticles industry.

## Material and methods

### Chemicals

All solvents were of HPLC grade, while those used in LC–MS–MS analysis were of MS grade. Rutin, gallic acid, and silver nitrate were purchased from Sigma-Aldrich (Schnelldorf, Germany).

### Plant material

*Punica granatum* leaves were collected from the botanical garden of Cairo University. Identity was verified by Prof. Mohammed El-Sayed, Horticultural Research Institute, Agriculture Research Centre, Ministry of Agriculture, Giza. A voucher specimen (No. PG002) has been deposited in the herbarium of the Faculty of Pharmacy, BUE, Cairo, Egypt.

### Preparation of extract and fractions

Five hundred gram of PL were extracted by maceration in 1 L of 70% ethanol for 48 h at room temperature, followed by filtration. The extraction process was repeated 3 times. The extracts were collected and dried under reduced pressure at 45 °C. The dried hydroalcoholic extract was suspended in distilled water and fractionated using solvents of increasing polarities, which are *n*-hexane, methylene chloride, ethyl acetate and *n*-butanol. Fractions were dried to yield 0.45 g, 9 g, 12 g and 10.5 g respectively.

### Spectrophotometric analysis of total polyphenols and flavonoids contents

Total polyphenols and flavonoids contents of ethyl acetate and *n*-butanol fractions in comparison to the crude extract were assayed using Folin-Ciocalteu method and aluminium chloride method respectively, that were reported by Attard^49^ and Herald et al.^50^, utilizing the microplate reader Fluostar Omega (BMG Labtech, Germany). Gallic acid and rutin were used as standards to calculate the total polyphenols content (mg GAE/g extract or fraction) and total flavonoids content (mg RE/g extract or fraction). The range of gallic acid concentrations to establish a calibration curve was from 7.8 to 500 μg/mL, while that of rutin was from 50 to 1,000 μg/mL.

### Isolation and identification of the major compound

The major phenolic compound in the PL extract was isolated by automated flash chromatography technique (Puriflash 4100 system—Interchim; Montlucon, France) with PDA–UV–Visible detector 190–840 nm and equipped with Puriflash column 30 C18 HP (20 bar). For system controlling and process monitoring, Interchim Software 5.0 was used. 10 g of the polyphenols rich fraction was dissolved in 50 mL of ethanol, then introduced into the column via dry loading technique using 10 g celite gel. Mobile phase was composed of 0.1% formic acid in water (Solvent A), and acetonitrile (Solvent B). The total run was for 210 min, and the gradient program was; 0–15 min (5–10% B), 15–135 min (10% B), 135–165 min (25% B), 165–180 min (45% B) and 180–210 min (100% B). The flow rate was 30 mL/min, and totally, 300 fractions were collected, each was with volume of 20 mL. The compound was precipitated as yellowish buff crystals from the fractions 20–80. The structure of the compound was elucidated by ^1^H and ^13^C NMR analyses that were recorded by Bruker Avance III HD FT-high resolution, Geramany- ^1^H-NMR (400 MHz), ^13^C-NMR (100 MHz) at Faculty of Pharmacy-Mansoura University.

### Standardization of the polyphenols rich fraction using Ultra performance liquid chromatography (UPLC) analysis

The polyphenols rich fraction was standardized. The experiment was carried out on Thermo Fisher UPLC Model Ultimate 3,000 (USA), equipped with PDA–UV–Visible light detector, on a column Hypersil GOLD (250 mm × 4.6 mm i.d.) and particle size 5 µm. Mobile phase was composed of 0.1% phosphoric acid in water as solvent A, and acetonitrile as solvent B, with constant flow rate at 0.7 mL/min. The gradient program was, 0–7 min (5–15% B), 7–10 min (15% B), 10–22 min (15–35% B), 22–35 min (35–100% B) and 35–40 min (100–5% B). Injection volume was 20 μL and column oven temperature was 30 °C. A calibration curve of the isolated major phenolic compound was established with concentrations range (31.25–1,000 μg/mL) and ethyl acetate fraction concentration was 5 mg/mL. All analyses were carried out in triplicate.

### LC–MS–MS analysis of the polyphenols rich fraction

The parameters of the LC–MS analysis were adjusted according to the method developed by Alfaifi et al.^51^. In details, the chromatographic separation was carried on Waters Acquity Xevo TQD system, on column Acquity BEH C18 100 mm × 2.1 mm column (p.s., 1.7 µm) (Waters, Ireland). Absolute ethanol was used to solubilize the sample at concentration of 1 mg/mL and filtered through a micropore filter of size 0.2 µm, the injection volume was 10 μL. The mobile phase system composed of Solvent A (0.1% formic acid in water) and Solvent B (0.1% formic acid in acetonitrile) at flow rate 200 μL/min with gradient elution: 0–4 min (15% B), 4–8 min (20% B), 8–30 min (55% B), 30–35 min (90% B) and 35–40 min (15% B). The high resolution mass spectra were recorded by Xevo TM triple-quadrupole tandem mass spectrometer with electrospray ionization (ESI) interface (Waters Corp., Milford, MA, USA) at mass ranges from 100 to 1,000 m/z, capillary voltage, 3.5 kV; detection at cone voltages, 20 V—95 V; radio frequency (RF) lens voltage, 2.5 V; source temperature,150 °C and desolvation gas temperature, 500 °C. Nitrogen was used as desolvation and cone gas at a flow rate of 1,000 and 20 L/h, respectively. System operation and data acquisition were controlled using Mass Lynx 4.1 software (Waters) .

### Green synthesis of AgNPs

AgNPs were synthesized by reduction of silver ions (Ag^+^) of silver nitrate solution to silver metal (Ag^o^). Briefly, 250 mL of silver nitrate solution at varying concentrations (1–5 mM) were heated to a temperature ranging from 60 to 80 °C, followed by addition of 50 mL of the plant material solution (hydroalcoholic extract, ethyl acetate fraction, or *n*-butanol fraction) at concentrations 0.5–5 mg/mL, with continuous stirring at 1,500 rpm for 1 hAfter that, AgNPs were pelleted by centrifugation at 10,000×*g* for 90 min at 4 °C, then washed thrice by deionized water, lyophilized and stored at − 18 °C for further analysis.

### Systematic optimization of the green synthesis process

Box-Behnken design was used to optimize the green synthesis process for production of AgNPs with the aid of Design Expert ver. 11.0 (Stat-Ease Inc., Minneapolis, USA). The independent variables for the optimization process were silver nitrate concertation, plant material concentration and temperature. Each variable was tested at three different levels; low (− 1), medium (0), and high (+ 1). A total of 14 trials were suggested by the selected design. Particle size and Polydispersity index (PDI) were analyzed as responses. After feeding the data in the design, mathematical modelling was carried out for analysis of results. Two factor interaction (2FI) process order was the best fitting model for the particle size response, while quadratic second order model was the suggested fitting model for the PDI response. After that the results were analyzed by ANOVA. The optimum conditions were identified by graphical optimization techniques and numerical desirability function.

### Characterization of AgNPs

#### UV–Visible spectral analysis

Surface plasmon resonance (SPR) bands of AgNPs were recorded on UV–Visible spectrophotometer device (Jasco-V630, Jasco Inc., MD, USA) at different time intervals during the green synthesis process. The wavelengths range of the spectral analysis was from 300 to 700 nm.

#### Fourier transform infrared spectroscopy (FTIR)

FTIR spectroscopy analysis for AgNPs was carried out on Vertex 70 FTIR, Brucker (USA). A thin film of the sample was allowed to form on KBr pellet and spectra were recorded.

#### Particle size analysis by light scattering technique

The mean particle size (diameter) and size distribution (PDI) of the synthesized nanoparticles were analysed with dynamic light scattering measurements at a scattering angle of 173°. Measurements were performed using a Zetasizer Nano ZS (Malvern Instruments, UK), where the dried nanoparticles were reconstituted in deionized water.

#### X-ray diffraction (XRD)

XRD measurement was carried out on X-ray diffractometer (Empyrean—Malvern Panalytical—Netherland), at X-ray power 40 kV and 30 mA and the spectrum was recorded by CuKα radiation with wavelength of 1.5406 Å in the 2θ range of 4°–80°, with a continuous scan type, step size (2θ) was 0.0200 and scan step time was 0.5 s.

#### Scanning electron microscopy (SEM)

Field emission SEM (Quattro S, Thermo scientific, USA) was used for the purpose of imaging of the synthesized AgNPs in order to study their shape and size. A small amount of AgNPs were placed on carbon coated copper grid. Then images were recorded at a magnifications × 160,000, × 480,000 and × 960,000.

#### Antimicrobial activity of the prepared AgNPs

Antimicrobial activity of the AgNPs were analysed by well diffusion method that was reported by Jyoti et al.^23^ as their AgNPs were synthesized using a plant extract and the particle sizes range was nearly the same as our findings. The analysis was carried out against G + ve bacteria (*Bacillus subtilis, Staphylococcus aureus,* and *Sarcina lutea*), G -ve bacteria (*Salmonella paratyphi*, *Escherichia coli,* and *Pseudomonas aeruginosa*) and fungi (*Candida albicans*). In each plate, five different concentrations of AgNPs were tested (0.05, 0.15, 0.25, 0.35 and 0.45 mg/100 µL), ethyl acetate fraction (0.45 mg/100 µL) and silver nitrate (0.45 mg/100 µL). The plates were incubated for 24 h at 37 °C, then the inhibition zone diameters were recorded.

## Data Availability

All data generated or analyzed during this study are included in this published article.
